# Acupuncture for amnestic mild cognitive impairment: A pilot multicenter, randomized, parallel controlled trial

**DOI:** 10.1097/MD.0000000000027686

**Published:** 2021-11-19

**Authors:** Jiayu Zhang, Xu Kuang, Chunzhi Tang, Nenggui Xu, Songhua Xiao, Lingjun Xiao, Shengwen Wang, Yu Dong, Liming Lu, Liang Zhang

**Affiliations:** a Medical College of Acu-Moxi and Rehabilitation, Guangzhou University of Chinese Medicine, Guangzhou, China; b Guangzhou Health Science College, Guangzhou, China; c Clinical Research Center, South China Research Center for Acupuncture and Moxibustion, Medical College of Acu-Moxi and Rehabilitation, Guangzhou University of Chinese Medicine, Guangzhou, China; d Department of Neurology, Sun Yat-sen Memorial Hospital, Sun Yat-sen University, Guangzhou, China; e Clinical Research and Data Center, South China Research Center for Acupuncture and Moxibustion, Medical College of Acu-Moxi and Rehabilitation, Guangzhou University of Chinese Medicine, Guangzhou, China.

**Keywords:** acupuncture, amnestic mild cognitive impairment, clinical trial

## Abstract

**Introduction:**

Patients with amnesic mild cognitive impairment (aMCI) are more likely to develop Alzheimer disease than corresponding age normal population. Because Alzheimer disease is irreversible, early intervention for aMCI patients seems important and urgent. We have designed a pilot multicenter, randomized, parallel controlled trial to assess the efficacy and safety of acupuncture on aMCI, explore the feasibility of acupuncture in the treatment of aMCI, so as to provide a reference for large-sample clinical trials in the next stage.

**Method:**

We designed a pilot multicenter, randomized, parallel controlled trial. This trial aims to test the feasibility of carrying out a large-sample clinical trial. In this trial, 50 eligible patients with aMCI will be included and allocated to acupuncture group (n = 25) or sham acupuncture group (n = 25) at random. Subjects will accept treatment 2 times a week for 12 weeks continuously, with a total of 24 treatment sessions. We will select 6 acupoints (GV20, GV14, bilateral BL18, bilateral BL23). For the clinical outcomes, the primary outcome is Montreal cognitive assessment, which will be assessed from baseline to the end of this trial. And the secondary outcomes are Mini-mental State Examination, Delayed Story Recall, Clinical Dementia Rating scale, Global Deterioration Scale, Activity of Daily Life, Alzheimer Disease Assessment Scale-Cognitive Section, brain magnetic resonance imaging, brain functional magnetic resonance imaging, and event-related potential P300, which will be assessed before and after treatment. In addition, we will assess the safety outcomes from baseline to the end of this trial and feasibility outcome after treatment. We will evaluate neuropsychological assessment scale (Montreal cognitive assessment, Mini-mental State Examination, Alzheimer Disease Assessment Scale-Cognitive Section) at 3 months and 6 months after treatment.

**Discussion:**

This pilot trial aims to explore the feasibility of the trial, verify essential information of its efficacy and safety. This pilot study will provide a preliminary basis for carrying out a larger clinical trial of acupuncture on aMCI in near future.

## 1 Introduction

With the world's population ages rapidly, Alzheimer disease (AD), the fourth main reason of death, will endanger more and more people's lives. World Health Organization predicts that the number of patients suffering from dementia will increase to 82 million in 2030 and 152 million by 2050. And AD is the commonest form of dementia.^[1]^ AD with high disability rate not only seriously reduces the quality of life of elderly patients, but also brings great economic losses and mental burden to their families. As there is no effective method to cure AD, early prevention, early diagnosis, and early treatment are particularly important.

Mild cognitive impairment (MCI) is progressive memory disorder similar to AD, which emphasizes the state of illness. Cognitive impairment includes not only memory impairment, but also other impairments, such as executive function, attention, language ability, and so on. According to the existence of memory impairment, Petersen and Negash^[2]^ divided MCI into 2 types: amnestic mild cognitive impairment (aMCI) and non-amnestic mild cognitive impairment, both of which can be further subdivided into single-region and multi-region. At present, aMCI is the most studied one, and most aMCI patients would develop AD, with 10% to 15% developing AD within 1 year, 40% developing AD within 2 years, 20% to 53% developing AD within 3 years, and 55% developing AD within 4 to 5 years, while the incidence of AD in the corresponding age normal population is only about 1% to 2% per year.^[3]^

The aMCI is considered to be the golden age to save memory impairment. In recent years, it has gradually become a research hotspot. Cognitive decline is the most prominent behavioral manifestation of aMCI patients. Unlike AD, aMCI patients retain a greater degree of cognitive ability to learn and use new skills, which makes them more suitable for early intervention. Because AD is irreversible, early intervention for aMCI patients seems important and urgent.^[4]^

At present, active intervention on MCI is an effective measure to delay the further decline of cognitive function. However, there is no consensus on which drugs or methods to intervene effectively, mostly in the experimental stage. Drug therapy for MCI includes cholinesterase inhibitors, estrogen replacement therapy, Ginkgo biloba preparations, non-steroidal anti-inflammatory drugs, and vitamin E. Current studies have shown that no drug has been found to be significantly beneficial for cognitive improvement or aMCI's transition to dementia. Most of the drugs belong to chemical preparations, which inevitably produce side effects on elderly patients. Non-drug treatment is mainly cognitive rehabilitation training, which is one of the methods to maintain the cognitive function of dementia patients, promote their maximum potential and make their condition relatively stable. The contents include: patient memory training, reality orientation, recalling and remembering the past, behavior management, and cognitive therapy, so as to improve the living quality of patients. But it has some shortcomings, such as limit efficacy, obvious cultural differences, poor compliance, and high treatment costs.

Recently, acupuncture becomes one of the most potential and widely recognized therapies to treat aMCI. It was showed that acupuncture combined with rehabilitation training can effectively improve the cognitive function of patients with MCI.^[5,6]^ Functional magnetic resonance imaging (fMRI) study^[7,8]^ of acupuncture treatment for elderly patients with MCI showed that acupuncture at Taixi acupoint can target activate the lesion brain area of MCI patients, namely the brain area closely related to cognition, memory, and emotion, and has a holistic regulatory effect on the related brain area.

Despite some evidence of acupuncture for aMCI emerging, methodological quality is generally not high enough to produce convincing and suggestive results.^[9]^ A rigorously designed randomized controlled trial (RCT) aims to explore the efficacy and safety of acupuncture on aMCI is warranted. Compared with the previous RCTs, the methodological design of this trial is very strict and the trial has the following innovations: The sham needle device will be used for the first time in the treatment of aMCI patients by acupuncture; The influence of lifestyle factors on aMCI patients’ memory will be strictly excluded; Data will be managed by data management system in order to avoid the interference of human factors on the clinical research results. This pilot study will preliminarily assess the efficacy and safety of acupuncture on aMCI, and explore the feasibility of acupuncture in the treatment of aMCI, so as to provide a reference for large-sample clinical trials in the next stage.

## 2 Method

### 2.1 Study design

**Figure 1:**
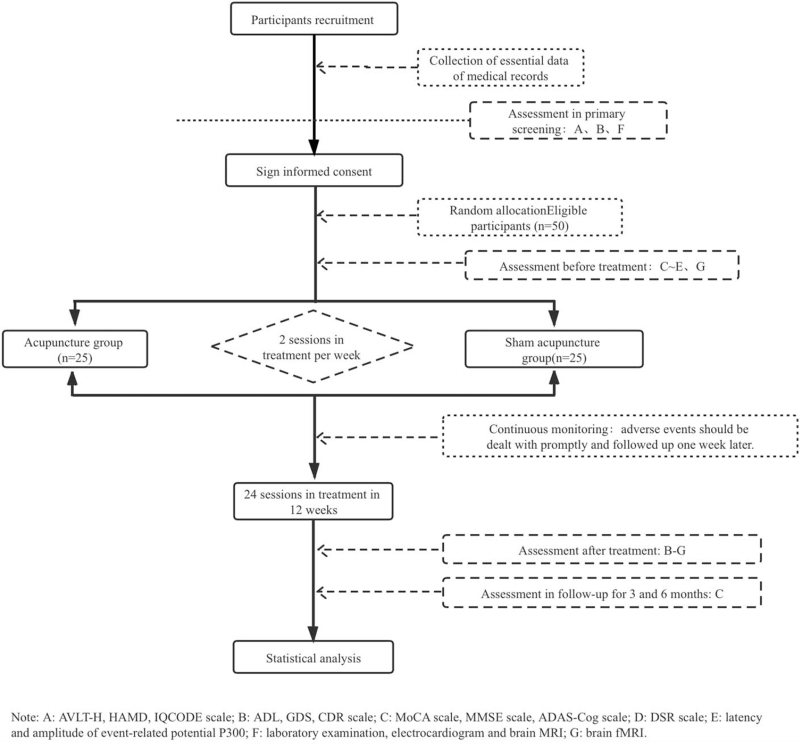
Flow chart the study.

The trial is a pilot multicenter, randomized, parallel controlled clinical trial. The clinical study will be carried out at least 3 clinical trial centers, including the First Affiliated Hospital of Guangzhou University of Chinese Medicine, Shenzhen Bao’an Traditional Chinese Medicine Hospital, and Guangzhou Huangpi Hospital. Subjects will be allocated to the test group or the control group at random for 2 treatment per week in 12 weeks, with a total of 24 sessions. The test group will receive acupuncture and conventional therapy, and the contrast group will receive sham acupuncture and conventional therapy. During the whole duration of the trial, subjects will be required not take other drugs for MCI, such as cholinesterase inhibitors, estrogen replacement therapy, Ginkgo biloba preparations, non-steroidal anti-inflammatory drugs, and vitamin E. Assessments will be conducted at pre-therapy, posttherapy, and follow-ups at 3, 6 months after the end of treatment. The flow chart of the study is shown in Figure 1. Time of outcome assessment and data collection is shown in Table 1.

**Table 1:** Time of outcome assessment and data collection.

	Study period				
	enrolment	Baseline	Posttherapy phase	Follow-up phase	
Timepoint	–1 week	0 weeks	12 weeks	3 months	6 months
Enrolment					
Eligibility screen	×				
Medical history	×				
Informed consent		×			
Allocation		×			
Interventions					
Acupuncture		×			
Sham acupuncture		×			
Assessments					
Clinical outcomes					
MoCA		×	×	×	×
MMSE		×	×	×	×
DSR		×	×		
CDR		×	×		
GDS		×	×		
ADL		×	×		
ADAS-cog		×	×	×	×
Brain MRI		×	×		
Brain fMRI		×	×		
Event-related potential P300		×	×		
Adverse events		×	×	×	×
Safety					
Safety assessment		×	×	×	×
Feasibility					
Treatment completion rate					×
Completion rate of aerobic exercise					×
Rate of drop-out					×
Response rate					×
Blinding test					×
Satisfaction					×

ADAS-cog = Alzheimer Disease Assessment Scale-Cognitive Section, ADL = Activity of Daily Life, CDR = Clinical Dementia Rating scale, DSR = Delayed Story Recall, GDS = Global Deterioration Scale, fMRI = functional magnetic resonance imaging, MMSE = Mini-mental State Examination, MoCA = Montreal cognitive assessment, MRI = magnetic resonance imaging.

### 2.2 Participants

Prospective participants will be diagnosed by geriatric psychologists or neuroscientists, who meet all inclusion criteria and did not fulfill any exclusion criteria. Research assistant will tell them the details about the study and ask them for some information about the eligibility criteria. Qualified participants who have interest in taking part in the study will be arranged to conduct some evaluations on their state and safety. After signing informed consent, participants will be assigned to 2 groups with different treatments.

### 2.3 Recruitment

Participants will be widely recruited by publishing paper recruitment advertisements on the spot and electronic recruitment advertisements on the Internet. Participants who have interest in taking part in the research will be screened in relevant test units. Research assistant will offer a written informed consent form to participants or their carers and tell them all detailed information and potential risks of the study. And participants or their carers will be witnessed to sign the informed consent by research assistant.

### 2.4 Diagnostic criteria

The diagnosis of aMCI in this study will be made by geriatric psychologists or neuroscientists, and the diagnostic criteria will be based on the relevant criteria in “The Diagnostic and Statistical Manual of Mental Disorders (5th Edition)”^[10]^:

1. Patients themselves, family members, or insiders provide chief complaints of memory impairment. Patients or insiders report that cognitive function has declined over the past year compared with previous ones.
2. The memory test (Auditory Verbal Learning Test-Huashan version Scale) score is 1.5 standard deviations lower than those of the normal comparisons matched for age and education.^[11]^
3. The general cognitive grading scale is mild abnormality, that is, the Global Deterioration Scale (GDS) is assessed as grade 2 to 3 or the Clinical Dementia Rating Scale (CDR) is scored as 0.5.
4. Daily living activities are intact or very slightly impaired, Activity of Daily Life (ADL) score = 20.
5. Not fulfilling the diagnostic criteria of dementia.

Patients who meet all of the above 5 criteria will be diagnosed as aMCI.

### 2.5 Inclusion criteria

Participants fulfilling all the following 5 items will be included:

1. Meeting the above aMCI diagnostic criteria.
2. Informant Questionnaire on Cognitive Decline in the Elderly Scale score is ≥51 and ≤53.
3. Aged 55 to 79 years, no gender limitation.
4. Adequate visual and auditory discrimination for neuropsychological testing.
5. Subjects participate voluntarily and sign informed consent.

### 2.6 Exclusion criteria

Participants fulfilling any of the following fourteen items will be excluded:

1. Education level <6 years.
2. Hamilton Depression Scale score is ≥17, pseudo-dementia caused by depression and other emotional abnormalities.
3. Patients with congenital mental retardation, patients with medical or neuropsychological state that can cause brain dysfunction, such as having a family history of dementia or having been diagnosed as dementia, psychosis, etc.
4. Patients with a history of cerebrovascular diseases, including ischemic cerebrovascular diseases, hemorrhagic cerebrovascular diseases, and neurological disorders such as hemiplegia, aphasia, etc.
5. Patients with secondary metabolic disorders caused by some endocrine, genetic and neurological diseases, patients with metabolic diseases induced by drugs.
6. Critically ill patients, patients with other central nervous injury diseases or history, such as brain trauma, encephalitis, epilepsy, tumor, infection, severe liver and kidney dysfunction, blood system diseases, central nervous system demyelination diseases and degenerative diseases of the central nervous system, etc.
7. Patients with a history of alcoholism and drug abuse, patients with long-term users of drugs that affect cognitive function, such as glucocorticoids, antipsychotics, sedatives and hypnotics, etc.
8. Patients who complain of snoring symptoms or whose family members state that they have snoring symptoms, with an Epworth sleepiness scale score ≥16,^[12,13]^ or diagnosed as sleep apnea syndrome by multiple physiological sleep tests (polysomnography).
9. Having participated in other research programs in the past 3 months.
10. Having received acupuncture treatment in the past 1 month.
11. Allergic to needles or alcohol.
12. Patients with syphilis or HIV positive.
13. Patients with abnormal vitamin B12 or thyroid dysfunction.

### 2.7 Withdrawal criteria

Participants fulfilling the following item will be withdrawn:

Occurrence of any serious adverse events (AEs): death or visceral function impairment caused by fainting during acupuncture treatment; stroke or accidental death during acupuncture treatment.

### 2.8 Interventions

Fifty eligible participants will be randomly divided into 2 groups. Both groups will receive conventional treatment (including basic drug therapy, cognitive rehabilitation training, diet and lifestyle guidance). On the basis of conventional treatment, the test group will be given acupuncture treatment, while the control group will be given sham acupuncture. They will be treated twice a week for 12 weeks. The time of each treatment wall be 30 minutes. Acupuncture venue is a separate closed-door treatment room. Only the acupuncturists and a subject will be present at the same time to ensure that different subjects will not guess the way of needling received by each other.

Acupuncturists of the study have registered Chinese Medicine Practitioners in China, who have been working in the clinic for at least 3 years and have a wealth of clinical experience in acupuncture practice. All of the acupuncturists will accept training before the study, which include the acupoints location, acupuncture operation skills, and communication skills. After the acupuncturists pass training test, they will be allowed to take part in the treatment of the study.

**Figure 2:**
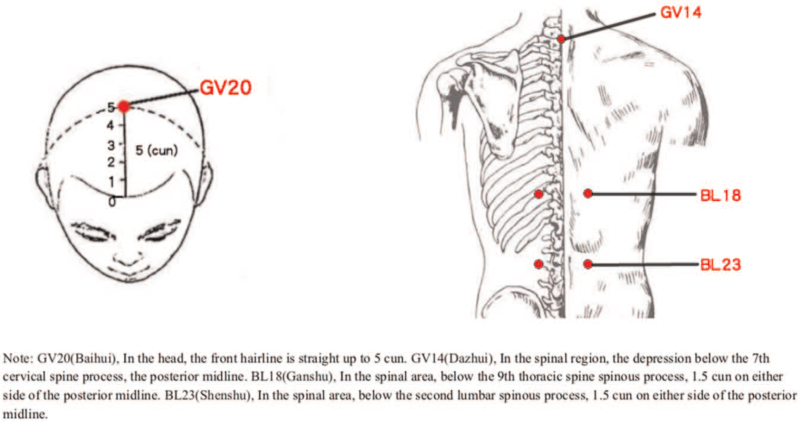
Locations of acupoints.

**Figure 3:**
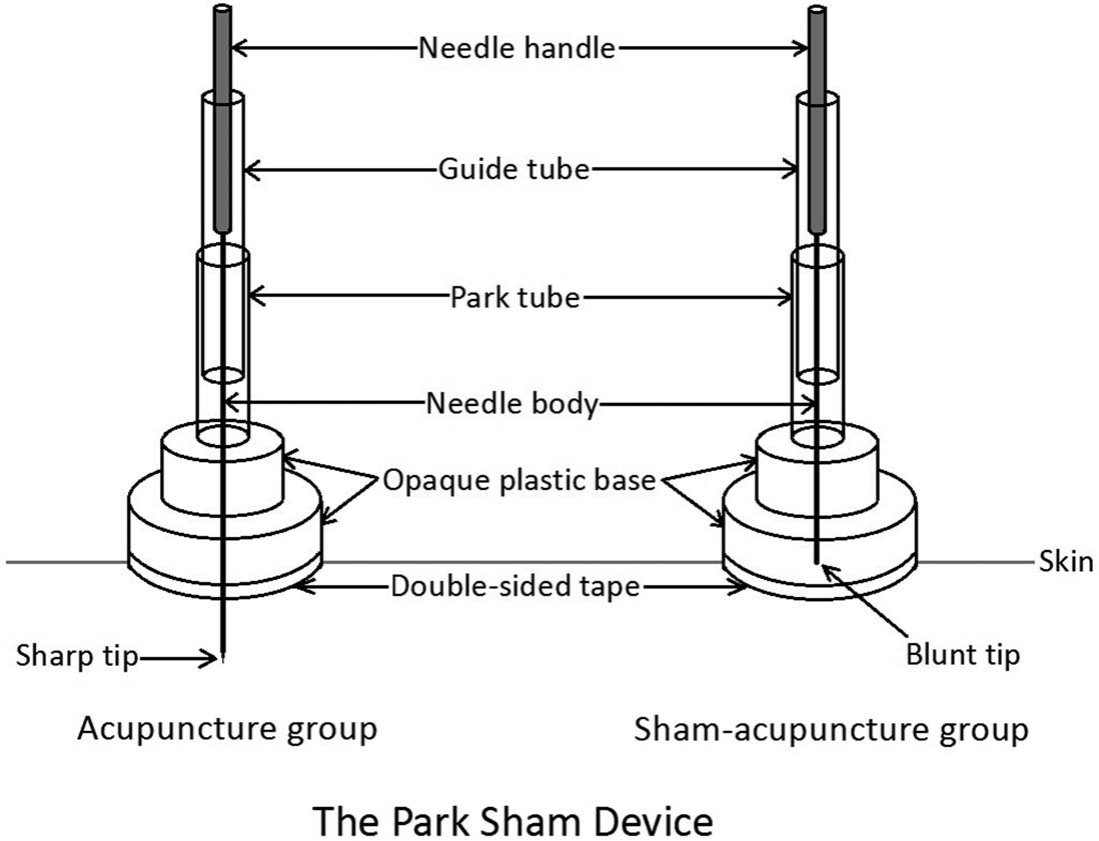
Park sham device.

In the test group, needles for acupuncture will be standard stainless steel, sterile, and disposable (Huatuo Medical Instruments Co. Ltd., Suzhou, China; 0.3 mm × 25 mm/0.3 mm × 40 mm), and electroacupuncture apparatus will be HANS-200E electroacupuncture apparatus. In the control group, needles for acupuncture will be placebo-needle with blunt-tip (Huatuo Medical Instruments Co. Ltd., Suzhou, China; 0.3 mm × 25 mm/0.3 mm × 40 mm), and electroacupuncture apparatus will be HANS-200E sham electroacupuncture apparatus without electrical pulse. In each group, Park Sham Device (ACUPRIME company) will be used, and the acupoints are same. The methods of acupuncture and acupoints are demonstrated in Table 2. The locations of acupoints are shown in Figure 2. The Park Sham Device is shown in Figure 3.

**Table 2:** Details of intervention.

	Intervention group	Control group
Acupoints	GV20, GV14, BL18, BL23	GV20, GV14, BL18, BL23
Angle of insertion	GV20, 15° GV14, BL18, 45° BL23, 90°	No insertion
Depth of insertion	15–25 mm	No insertion
Needle type	Stainless steel needle (Suzhou Medical Supplies Factory Co., Ltd.)	Blunt-tip needle (Suzhou Medical Supplies Factory Co., Ltd.)
Needle sensation	With de-qi sensation	Without de-qi sensation
Electric stimulation	Needle on BL18, BL23 connected to a HANS-200E electroacupuncture apparatus, whose waveform is dilatational wave with alternating frequency of 2 Hz and 100 Hz	Needle on BL18, BL23 connected to a HANS-200E sham electroacupuncture apparatus, without electrical pulse
Frequency and duration	Two times per week for 12 weeks	Two times per week for 12 weeks

### 2.9 Conventional treatment

#### 2.9.1 Basic treatment

Basic treatment mainly includes blood pressure management, blood glucose control, and other supportive treatment.

#### 2.9.2 Cognitive rehabilitation training

Through the Software System of Brain Function Information Management Platform,^[14]^ doctors will adopt a one-to-one way to conduct cognitive rehabilitation training independently. The cognitive module in the system includes cognitive games with different contents and difficulty, which train the subjects’ attention (including attention retention, attention distribution, and attention span), self-control, reaction speed, motor perception, and memory (including the memory of space, face, and number). Each training time is 30 minutes. Treatment will be performed 2 times a week for 12 weeks, with a total of 24 times.

#### 2.9.3 Diet and lifestyle guidelines

Refer to Neal D. Barnard et al's “Diet and Lifestyle Guidelines for AD Prevention” published in Neurobiology of Aging.

(1) Try to reduce the intake of saturated and trans fats. Dairy products, meat, and some oils (e.g., coconut oil and palm oil) contain a lot of saturated fats. Snack cakes and fried foods contain a lot of trans fats.
(2) Replace meat and dairy products with green leafy vegetables, legumes, fruits, and whole grains as staple foods of daily diet.
(3) Vitamin E is taken through food rather than vitamin supplements, such as seeds, nuts, green leafy vegetables, and whole grains. The recommended dietary allowance of vitamin E is 15 mg a day.
(4) A cumulative daily intake of 2.4 mg B12 is achieved through food or oral B12 tablets.
(5) Avoid eating foods or herbal preparations, which contain iron and copper.
(6) Avoid using utensils and cookers containing aluminum, and avoid eating foods containing antacids, baking powder, or other foods containing aluminum.
(7) Insist on moderate aerobic exercise. Walk fast at least 3 times a week for 40 minutes each time.

#### 2.9.4 Acupuncture group

Subjects in the group will accept acupuncture and conventional treatment. On the basis of in-depth study on aMCI and experience of acupuncture, Tongdu Tiaoshen Acupuncture, namely Baihui (GV20), Dazhui (GV14), and bilateral Ganshu (BL18), and bilateral Shenshu (BL23) will be used as therapeutic acupoints. Acupoint location is on the basis of the national standard of the People's Republic of China “Name and Location of Acupoints” (GB/T 12346-2006, issued from September 18, 2006-12-01). The base of Park Sham Device will be adhered to the skin of the acupoint. We will insert the needle into the tube and tap the needle handle to make the needle tip pierce the skin. Shenshu (BL23): continue to insert deep into the acupuncture point vertically; Baihui (GV20), Dazhui (GV14), Ganshu (BL18): remove the tube and base, remaining the tape and continue to insert the needle obliquely. We will insert the needles into the skin with the depth of 15 to 25 mm and manipulate the needle manually (containing lifting, thrusting, and rotating) until the participants states needling sensations (De-qi sensation). Then the needle handle will be rotated continuously for 2 minutes at a frequency of about 200 times per minute, with small amplitude, fast speed and uniform speed. The needles on BL18 and BL23 will be connected to a HANS-200E electroacupuncture apparatus, whose waveform is dilatational wave with alternating frequency of 2 Hz and 100 Hz. Needles will be retained for 30 minutes before removing.

#### 2.9.5 Sham-acupuncture group

Subjects in this group will accept sham acupuncture and conventional treatment. The needles will not insert into the skin, and acupoints are the same as test group. A non-invasive placebo control, namely sham blunt-tip needle, will be used in this trial. The base of Park Sham Device will be adhered to the skin of the acupoint. We will insert the sham needle into the tube and tap the needle tip to make the needle touch the acupoint skin but not pierce the skin. The needle on BL18 and BL23 will be connected to a HANS-200E sham electroacupuncture apparatus without electrical pulse. Needles will be also retained for 30 minutes before removing.

### 2.10 Outcome measurements

#### 2.10.1 Clinical outcomes

We will evaluate the neuropsychological assessment scale (Mini-mental State Examination [MMSE], Montreal cognitive assessment [MoCA], CDR, Delayed Story Recall, GDS, ADL, Alzheimer Disease Assessment Scale-Cognitive Section [ADAS-cog]), brain cranial magnetic resonance imaging (MRI) and brain functional cranial fMRI, latency and amplitude of event-related potential (ERP) P300 before and after treatment. This incidence rate of acupuncture AEs will be counted. We will evaluate neuropsychological assessment scale (MMSE, MoCA, ADAS-cog) at 3 months and 6 months after treatment.

#### 2.10.2 Primary outcome

##### 2.10.2.1 Montreal cognitive assessment

The MoCA contains 8 items: visuospatial and executive function, denominating, short-term memory, concentration, language, abstraction, delayed memory, and orientation. Total points range from 0 to 30. If participants have not been educated for more than 12 years, their scores will be increased by 1 point on their score (if <30). If the total score is less than 26, it represents cognitive decline.^[15]^ The lower the score, the more significant the cognitive impairment.

#### 2.10.3 Secondary outcomes

##### 2.10.3.1 Mini-mental State Examination

The MMSE evaluates 10 domains of cognitive function which includes orientation, short-term memory, attention and calculation, delayed memory, language and ability to draw a complex polygon etc. Total points range from 0 to 30. The lower the scores, the more significant the cognitive impairment.

##### 2.10.3.2 Delayed Story Recall

The Delayed Story Recall is to ask the subjects to listen to and retell the 2 stories first, at this time, no score will be given. After 30 minutes, the subjects retell the 2 stories, and then began to scoring. One point for each keyword, with a total point range from 0 to 40. The lower the score, the more serious the cognitive impairment.

##### 2.10.3.3 Clinical Dementia Rating scale

The CDR includes 3 cognitive items and 3 functional items, namely, memory, orientation, discretion/ability to solve problems, social affairs, family/interest, and personal care. Each item is grated as 5 level, namely, 0 point represents no impairment, 0.5 point indicates questionable impairment, 1 point indicates mild impairment, 2 points indicates moderate impairment, and 3 points indicates severe impairment. And there are 2 methods of scoring for CDR, including global score (GS) and sum of boxes (SOB) score.^[16]^ If CDR-GS is 0.5, it represents questionable dementia or MCI.^[17,18]^ If CDR-SOB is 0.5 to 4.0, it represents MCI.^[19,20]^ Higher score indicates more severe the cognitive impairment.

##### 2.10.3.4 Global Deterioration Scale

The GDS assesses the severity of cognitive decline over 7 stages, that is, stage 1 shows no cognitive impairment, stage 2 shows very mild cognitive impairment, stage 3 shows mild cognitive impairment, stage 4 shows moderate cognitive impairment, stage 5 shows moderate severe cognitive impairment, stage 6 shows severe cognitive impairment, stage 7 shows very severe cognitive impairment.^[21]^

##### 2.10.3.5 Activity of Daily Life

The ADL assesses 20 domains of activities, which includes physical self-maintenance scale and instrumental activities daily of life. Physical self-maintenance scale includes 6 basic activities: toilet, feed, dressing, grooming, physical moving, and bath, and instrumental activities daily of life includes 8 instrumental activities: using telephone, going shopping, preparing food, doing housework, doing the laundry, transportation, taking medicine, ability to handle finances.^[22]^ The scale is divided into 4 grades: 1 = self-care; 2 = have some difficulties; 3 = need help; 4 = unable to complete. The total score ranged from 20 to 80. If the score of single item is 1, it is normal; if the score of single item is 2 to 4, it is functional decline. If the total score = 20 points, it indicates absolutely normal; if the total score is >20, it indicates different levels of functional decline. The higher the score, the worse the ability to live.

##### 2.10.3.6 Alzheimer Disease Assessment Scale-Cognitive Section

The ADAS-cog includes 12 cognitive domains, containing recall of word, recognizing word, constructional practice, orientation, denominating, commands, ideational practice, recall test commands, verbal ability, looking for words, comprehension, and concentration. The total scores ranges from 0 to 75. The higher the score, the poorer the cognitive impairment.^[23,24]^

##### 2.10.3.7 Brain magnetic resonance imaging

MRI structural imaging can show different brain lesions, which is helpful for the etiological diagnosis and monitoring of the progress of MCI. The most common local brain changes in aMCI are atrophy of the hippocampus and the entorhinal cortex.^[25]^ Besides, atrophy of hippocampus and entorhinal cortex are reliable indicators of the transformation of aMCI to AD.^[26,27]^ Other brain changes on MRI in patients with MCI include decreased cortical gray matter, enlarged ventricles, and increased white matter high signal intensity.^[27]^

##### 2.10.3.8 Brain functional magnetic resonance imaging

Cerebral activation can be researched by Blood-Oxygen-Level-Dependent signal of fMRI. Blood-Oxygen-Level-Dependent signal would make differences when there are pathological changes associated with neurodegenerative disease.^[28–30]^ Default mode network is a focus network for the study of cognitive function by using resting-state functional MRI. It includes the posterior cingulate/anterior cuneiform lobe, medial prefrontal cortex, hippocampus, angular gyrus, and ventral anterior cingulate gyrus,^[31]^ which are related to cognitive function, executive function, and situational memory. resting-state functional MRI in aMCI patients is mainly manifested as the gradual weakening of functional connections in the default mode network of brain regions, such as hippocampus and medial prefrontal lobe.^[32]^

#### 2.10.4 Event-related potential P300

ERP are electric signals from the brain, which relate to miscellaneous cognitive function, like selective concentration, memory, or discretion.^[33–35]^ Component of P300 is the research hotspot of ERP and the numerous clinical P300 studies^[36–42]^ show that the component can be used as an index of cognitive function. The prolonged latency and declining amplitude of P300 shows cognitive impairment.

#### 2.10.5 Adverse events of acupuncture

The incidence rate of acupuncture's AEs during treatment will be counted. Clinical symptoms and related laboratory examination results of acupuncture's AEs will be recorded to judge their grades. Follow-up visits after AEs will be recorded.

### 2.11 Safety

We will evaluate vital signs, blood routine, urine routine, liver and kidney function, coagulation function, electrocardiogram, and clinical symptoms and related laboratory examination results of acupuncture's AEs at pre-therapy and posttherapy.

### 2.12 Feasibility

In order to evaluate the feasibility of carrying out RCTs with large samples in the next stage, we set the following indicators in this pilot trial. The feasibility outcomes are demonstrated in Table 3.

**Table 3:** Feasibility outcomes.

Feasibility outcomes	Evaluation criteria
1. Proportion of participants who completed the required 12-week acupuncture treatment and cognitive rehabilitation training program	If ≥80% participants completed, proceed; if 50%–80% participants completed, modify protocol; if <50% participants completed, do not proceed.
2. Proportion of participants who completed a 12-week aerobic exercise program	If ≥80% participants completed, proceed; if <80% participants completed, modify protocol.
3. Rate of drop-out during treatment	If rate of drop-out ≤20%, proceed; if rate of drop-out is 20%–50%, modify protocol; if rate of drop-out <50%, do not proceed.
4. Response rate	Response rate of subjects to the collected indicators will be evaluated at pre-therapy, posttherapy, 3 months and 6 months after treatment.
5. Blinding test	All the participants will be tested for the success of blinding after the final treatment session. They will be asked to judge whether they have received real acupuncture or sham acupuncture.
6. Satisfaction	Participants’ satisfaction with the intervention will be ranked on a 5-point scale, each point is very satisfied, satisfied, neutral, dissatisfied, and very dissatisfied.
7. Average number of eligible subjects recruited in each center per month	Each center separately counts the number of eligible participants.
8. Enrollment rate	Proportion of participants who were last included in the study among all eligible participants.

### 2.13 Sample size

Based on the RCT report published by Jiang et al^[43]^ in 2016, the sample size was estimated with MoCA as the main outcome. After 12 weeks of treatment, the difference of MoCA between pre-therapy and posttherapy in the conventional rehabilitation group (without acupuncture) was 2.83 ± 1.13, and that of MoCA between pre-therapy and posttherapy in acupuncture group was 1.33 ± 1.20. The test level was set at *α* = 0.05 and *β* = 0.1. By comparing the differences between 2 independent samples, PASS 2008 software was used to calculate that the required sample size of each group was 13 cases, and 26 cases in both groups.

To sum up, considering that the follow-up loss rate is 20%, the sample size should be 26/(1 – 0.2) = 33 cases. Considering that we have enough research capacity to fulfill the expected research tasks, we intend to expand the sample size to a total of 50 cases, 25 cases in each group.

### 2.14 Randomization and allocation concealment

The stratified random allocation operation of experimental group and control group will be completed by the researcher of the Clinical Research Center, South China Research Center for Acupuncture and Moxibustion, Guangzhou University of Chinese Medicine. The programming and randomized operation will be performed by PROC PLAN of SAS 9.2. The results of random allocation will be distributed through the central random allocation system in the internet.

### 2.15 Blinding

Patients will be notified that they will be received acupuncture treatment or acupuncture-like simulation treatment, which is randomly allocated. And they will be required to close their eyes during treatment. Only the acupuncturists will be aware of the detailed grouping. Subjects and other investigators (like data analysts, outcome evaluators, and statisticians) will not know group assignments. All investigators will receive trial training before the study starts to ensure the success of the implementation of blind method.

### 2.16 Blinding test

**Figure 4:**
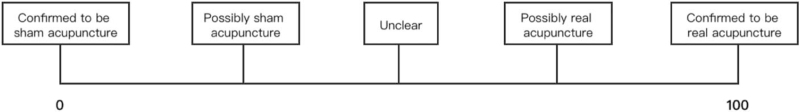
Blinding success assessment scale.

All subjects will be tested for the success of blinding after the final treatment session. They will be asked to judge whether they have received real acupuncture or sham acupuncture. In the scale of 0 to100, 0 is “confirmed to be sham acupuncture”, 100 is “confirmed to be real acupuncture”. After scoring, the results will be collected and statistically analyzed by the research team. The blinding success assessment scale is shown in Figure 4.

### 2.17 Safety and adverse events

If participants are considered to have undesirable effect after acupuncture treatment, they should discontinue treatment and tell acupuncturists. Acupuncturists will diagnose and cure the AE, and other investigator will contact them to fill in the AE form and AE record log. AEs may occur during acupuncture, such as fainting, hematoma, bent needle, stuck needle, broken needle, infection, etc. If any relevant AE occurs during the study, it will be dealt with immediately.

Participants and acupuncturists should report all of the AEs (include all discomfort, symptoms, or illness occurring during the study). All detailed information of AEs (include occurrence time, symptoms, duration, severity, treatment measures, and duration) will be reported in the case report forms (CRFs). In case of serious AE, it will be reported to the lead unit and the state administration of traditional Chinese medicine within 24 hours. One week after symptomatic treatment, follow-up will be conducted through outpatient service, telephone, or visit. We will analyze the impact of all AEs after this trial finishes.

### 2.18 Data management

All data will be input into a pre-designed, password protected dataset by someone who will not know the group assignment. In order to protect the subjects’ privacy, we will adopt codes and initials to mark the subjects’ personal information. Specified investigators will monitor the quality of online CRFs completion. Only outcome evaluators can access CRFs and will carry out double-data entry with any amendment or transformation of written data in subjects’ CRFs documented and dated. The clinical supervisor will monitor the data and study progress at least once a day.

### 2.19 Statistical analysis

The statistical tests involved in this study will include unilateral and bilateral tests with a significance level of alpha = 0.05. Statistical analysis will be completed by SPSS 22.0 software (International Business Machines Corp., Armonk NY).

Effect analysis will involve a population consisting of per-protocol subject analysis and intention-to-treat analysis. We will adopt a repeated measures design approach to compare the treatment outcomes (MoCA, MMSE, and ADAS-cog) over time between 2 groups. The model will be built to distinguish the intervention effect and the time effect. Among-group differences at each assess time point will be further tested by student *t* test. Categorical variables, containing categorical baseline variables and incidence of AEs, will be analyzed by a chi-square (χ^2^) or Fisher exact test.

### 2.20 Handling of missing data

If the missing value is less than 5%, a full case analysis without calculating missing date will be adopted. If the missing value exceeds 5%, Little test will be adopted. If Little test showed that the full case data set is a random sample, the full cases without calculating missing date will be continued analyzing. If Little test showed that the full case data set is not a random sample, the point estimation and 95% confidence interval will be reported, with a worst- and best-case scenario imputation for the missing date. If the worst- and best-case analysis reach the same conclusion, we will not carry out multiple imputations. Or else, under the assumption of randomly missing value, multiple imputations will generate 10 estimated data sets. The pooled intervention effect and 95% confidence interval of the analysis of every data set after multiple imputation will constitute the results of the trial.

### 2.21 Quality control

Before the experiment, all subjects participating in this study will receive the same training, detailed description of the study operation process, explanation of acupuncture related knowledge, and examination characteristics of ERP P300, MRI, and fMRI. All assessment will be conducted by a team led by a qualified professional evaluator. Researchers who participate in efficacy evaluation must have a certificate of medical practitioner and will not take part in the treatment of participants. All researchers will be trained and assessed by acupuncture physicians above the associate chief physician on standard operating procedure (including all kinds of questionnaire application methods, acupuncture methods, etc). In each research center, participants will be fixed as the same acupuncturist to perform the operation. Special independent supervisors will be set up in each research center. Each participant will be given a medication history book. The researchers will instruct participants to keep detailed records of all specific participant conditions during the study, including drug name, drug dosage, time of taking drug, and possible symptoms after taking drug. All data are entered into the data management system.

## 3 Discussion

Acupuncture therapy has been used in China for more than 2000 years^[44]^ and has been generally practiced in Western countries in recent years.^[45]^ Recently, despite some evidence of acupuncture for aMCI emerging, methodological quality is generally not high enough to produce convincing and suggestive results. We design a rigorous RCT, using the control group method to decline bias of test and ensure the dependability of the RCT. And the study is a significant RCT aimed to treat aMCI.

This RCT has some improvement compared with previous trials. Firstly, it is a problem for the acupuncture investigators how to select the acupuncture points.^[46]^ According to our in-depth study on aMCI and experience of acupuncture, Tongdu Tiaoshen Acupuncture, namely Baihui (GV20), Dazhui (GV14), and bilateral Ganshu (BL18), and Shenshu (BL23) will be used as therapeutic acupoints. Secondly, we select the MoCA as the primary outcome. Multiple guidelines^[27,47]^ suggest the MoCA as the primary outcome of aMCI, and many aMCI studies adopted the MoCA as an assessment outcome. Finally, we have set a control group to realistically examine recruitment, randomly allocation, complement of interventions, and blinding evaluation.^[48]^ Nowadays, placebo contrast for acupuncture contains non-acupoint, superficial, and sham acupuncture.^[49–51]^ Some people think efficacy of acupuncture is controversial, as acupuncture takes effect primarily by a placebo result, or the controls of sham acupuncture maybe not absolutely non-effective.^[52–54]^ In the study, control group will accept sham acupuncture, whose needles will not insert into the skin. In each group, Park Sham Device will be used, and the acupoints are same. The designs will help reduce non-specific influence in the sham acupuncture group. Besides, acupuncture venue is a separate closed-door treatment room. Only the acupuncturists and a subject will be present at the same time to ensure that different subjects will not guess the way of needling received by each other.

Nevertheless, the study still faces some challenges. Firstly, the screening criteria for subjects in this study are very strict to ensure the homogeneity of subjects’ baseline and the validity of test results. Therefore, it may not be possible to recruit a large number of eligible subjects in a short period of time. We will summarize the experience after preliminary test, so as to expand the sample size and carry out the formal experiment in the next stage. Secondly, it is almost impossible to implement blind method for acupuncturists and it is inevitable that acupuncturists will know the treatment. Thirdly, in China, especially in Guangdong, many elderly people have received acupuncture treatment and know the feeling of being needled, so it is not easy to blind the subjects in the study. Some subjects may guess whether they are receiving real acupuncture treatment or not. To ensure the smooth implementation of blinding, participants in the 2 groups will use Park Sham Device when receiving acupuncture, and all participants are required to close their eyes during the whole course of treatment. In order to standardize the use of these false needle devices, all acupuncturists participating in the treatment will receive unified training and assessment before the start of the study, so as to ensure the consistency of the needle manipulation process and stimulation intensity. Fourthly, subjects will spend a lot of time on treatment because each treatment takes at least 1 hour and lasts for 12 weeks. Therefore, we anticipate that the compliance of subjects may not be high enough. In order to improve the compliance of subjects, we keep in touch with the subjects and at least 1 family member of the subjects. Let them realize the importance of intervening in aMCI by explaining the relationship between aMCI and AD in detail. In addition to the subjects, we will inform the family members of the research process in detail, so that the family members would urge the subjects to complete the treatment process. Meanwhile, we will give some compensation incentives to the participants.

In order to reach our goals, we will try to make every step standard, such as selection of acupoints, manipulation of acupuncture, needle apparatus, and needle apparatus’ clinical experience. We look forward to that the study will offer strong positive proof for acupuncture on aMCI.

### 3.1 Trial status

This trial was registered on 27 February 2019 (registration number ChiCTR1900021557, protocol version number F3.0). At present, the clinical study is underway. This trail started on July 2019 and the expectant finished date of this trial is June 2020.

## Acknowledgments

We would like to express gratitude to the staff of South China Research Center for Acupuncture and Moxibustion of Guangzhou University of Chinese Medicine, the First Affiliated Hospital of Guangzhou University of Chinese Medicine, Shenzhen Bao’an Traditional Chinese Medicine Hospital and Guangzhou Huangpi Hospital.

## Author contributions

The trial was designed and developed by LZ and LML. The manuscript was drafted by JYZ and XK. The protocol was carefully revised and edited by CZT and NGX, SHX, LJX, YD, and SWW contributed to the discussion. All authors read and approved the final manuscript.

**Formal analysis:** Liming Lu, Liang Zhang.

**Investigation:** Nenggui Xu, Shengwen Wang.

**Methodology:** Songhua Xiao.

**Project administration:** Chunzhi Tang, Lingjun Xiao.

**Visualization:** Yu Dong.

**Writing – original draft:** Jiayu Zhang, Xu Kuang.

**Writing – review & editing:** Xu Kuang.
